# Lysosomal lipid hydrolysis provides substrates for lipid mediator synthesis in murine macrophages

**DOI:** 10.18632/oncotarget.16673

**Published:** 2017-03-29

**Authors:** Stefanie Schlager, Nemanja Vujic, Melanie Korbelius, Madalina Duta-Mare, Juliane Dorow, Christina Leopold, Silvia Rainer, Martin Wegscheider, Helga Reicher, Uta Ceglarek, Wolfgang Sattler, Branislav Radovic, Dagmar Kratky

**Affiliations:** ^1^ Institute of Molecular Biology and Biochemistry, Medical University of Graz, Graz, Austria; ^2^ Boehringer Ingelheim, Vienna, Austria; ^3^ Institute of Laboratory Medicine, Clinical Chemistry and Molecular Diagnostics, University Hospital Leipzig, Leipzig, Germany; ^4^ LIFE-Leipzig Research Center for Civilization Diseases, University of Leipzig, Leipzig, Germany; ^5^ BioTechMed-Graz, Graz, Austria

**Keywords:** lysosomal acid lipase, LAL-D, lysosome, eicosanoids, lipid mediator, Immunology and Microbiology Section, Immune response, Immunity

## Abstract

Degradation of lysosomal lipids requires lysosomal acid lipase (LAL), the only intracellular lipase known to be active at acidic pH. We found LAL to be expressed in murine immune cells with highest mRNA expression in macrophages and neutrophils. Furthermore, we observed that loss of LAL in mice caused lipid accumulation in white blood cells in the peripheral circulation, which increased in response to an acute inflammatory stimulus. Lal-deficient (-/-) macrophages accumulate neutral lipids, mainly cholesteryl esters, within lysosomes. The cholesteryl ester fraction is particularly enriched in the PUFAs 18:2 and 20:4, important precursor molecules for lipid mediator synthesis. To investigate whether loss of LAL activity affects the generation of lipid mediators and to eliminate potential systemic effects from other cells and tissues involved in the pronounced phenotype of Lal-/- mice, we treated macrophages from Wt mice with the LAL-specific inhibitor LAListat-2. Acute inhibition of LAL resulted in reduced release of 18:2- and 20:4-derived mediators from macrophages, indicating that lipid hydrolysis by LAL is an important source for lipid mediator synthesis in macrophages. We conclude that lysosomes should be considered as organelles that provide precursor molecules for lipid mediators such as eicosanoids.

## INTRODUCTION

Lysosomes are the main cellular organelles responsible for degradation of material derived from endosomal and autophagosomal pathways. Receptor-mediated endocytosis enables whole lipoprotein particle uptake involving endosome formation. After fusion with a lysosome, degradation products are provided as a source of energy or as substrates for anabolic processes of the cell [1]. During autophagy, intracellular digestion of damaged organelles, misfolded proteins, and unnecessary cellular material is initiated by the formation of a sequestering membrane generating an autophagosome. The fusion of the autophagosome with a lysosome results in an autolysosome, which degrades autophagic cargo [2]. Cytosolic lipid droplets may also be subjected to autophagic degradation in a process termed lipophagy [3]. Lysosomal acid lipase (LAL), the only known intracellular lipase active at acidic pH (pH 4-5), degrades cholesteryl esters (CEs) and triglycerides (TGs) in lysosomes [4]. It has been shown recently that LAL is also able to degrade retinyl esters [5].

In humans, LAL deficiency (LAL-D) causes a severe lysosomal storage disorder characterized by an increased accumulation of neutral lipids in multiple organs, including liver, spleen, adrenals, bone marrow, lymph nodes, and intestine [6, 7]. LAL-D patients have elevated LDL-cholesterol concentrations, reduced HDL-cholesterol levels and develop atherosclerosis [6], indicating a critical role for LAL in cellular lipoprotein and lipid metabolism. Furthermore, multiple organs, particularly liver and intestine, suffer from progressive and massive accumulation of lipid-laden macrophages. The importance of macrophages as the central regulators of systemic inflammation and the LAL-D phenotype is underlined by the general amelioration of the condition with favorable outcomes upon bone marrow transplantations from healthy donors [7].

LAL knockout (Lal-/-) mice suffer from severe hepatosplenomegaly, single enlargement of a mesenteric lymph node, systemic inflammation, fat tissue depletion, and lysosomal accumulation of CE and TG in various cells and tissues [8]. The absence of LAL impairs differentiation of myeloid progenitors. This leads to a systemic elevation of myeloid-derived suppressive cells, which are involved in tumor progression and alter the development, maturation, and functionality of T cells [9]. The critical role of macrophages in the pathophysiology of LAL-D was highlighted by partial rescue of the phenotype upon macrophage-specific expression of the enzyme in Lal-/- mice [10]. Furthermore, multiple evidence demonstrated the importance of lysosomal lipid processing in the regulation of cholesterol homeostasis [11], macrophage polarization, and inflammatory responses [12].

Fatty acids (FAs) are important energy sources, structural components of cell membranes, and signaling molecules. FAs directly affect immune cell activation by acting as ligands for extracellular (e.g. toll-like receptors, G-protein coupled receptors) and intracellular binding and transport proteins (e.g. fatty acid transport proteins, fatty acid binding proteins, and nuclear receptors). In addition, FAs such as eicosapentaenoic acid (20:5), docosahexaenoic acid (22:6), and arachidonic acid (20:4) are important precursors for the synthesis of biologically active lipids. Once liberated from the cell, these FAs are metabolized into lipid mediators through four different pathways: the COX, LOX, cytochrome P450 (CYP450), and the non-enzymatic oxidation pathway [13]. Phospholipids from distinct subcellular compartments including the ER, phagosome, nuclear envelope, and lipid droplets have been described to act as cellular pool for precursor FAs (reviewed in [14]). Recent studies revealed that also TG can act as source of precursor FAs. In these studies we showed that adipose triglyceride lipase (ATGL)-dependent mobilization of precursor molecules from the TG-rich lipid droplet pool of neutrophils and mast cells regulates the release of lipid mediators, including eicosanoids [15, 16]. Due to the importance of endosomal and autophagosomal pathways in FA release, we hypothesized that FAs derived from LAL-mediated lysosomal hydrolysis may be another important source to provide substrates for the generation of lipid mediators.

## RESULTS

### LAL is expressed in murine immune cells

**Figure 1:**
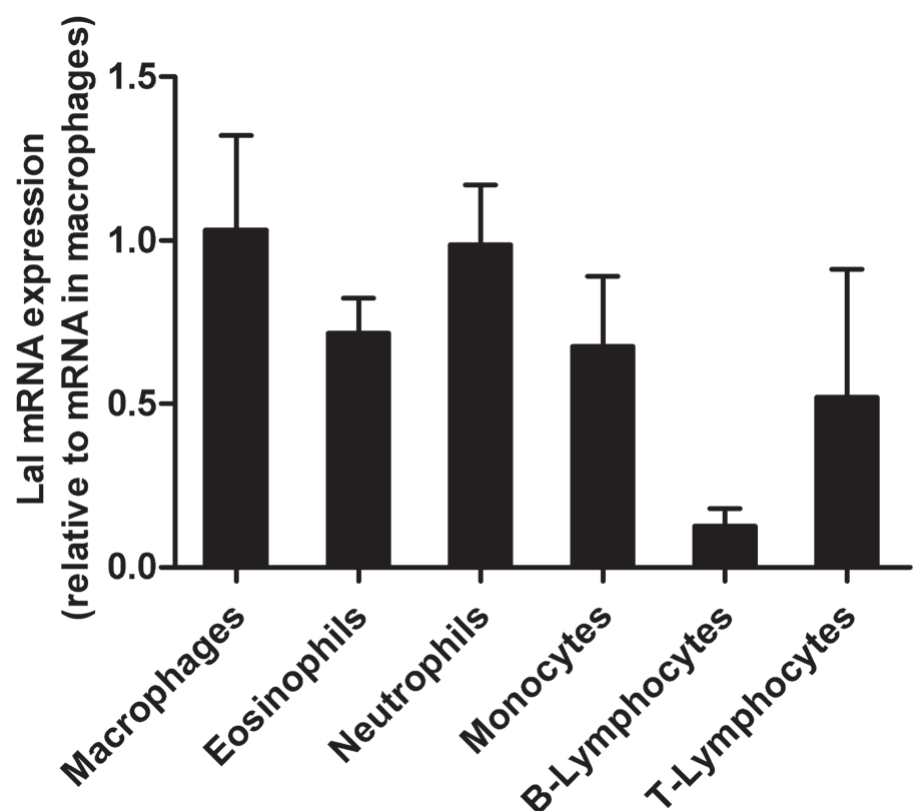
Lal mRNA expression in Wt immune cells Total RNA of flow-sorted immune cells was isolated and Lal mRNA expression was analyzed by quantitative real-time PCR relative to the expression of Gapdh as housekeeping gene. Transcript expression levels are shown as fold change relative to mRNA levels in macrophages and represent means + SD (*n* = 4).

We first determined expression level of LAL in various immune cells. We used fluorescence-activated cell sorting to distinguish between Wt immune cells, specifically neutrophils, monocytes, and macrophages (derived from thioglycolate-elicited lavage). Since the lavage contains only 1-2 % of T- and B-lymphocytes, we isolated these cells from peripheral blood. We assessed Lal mRNA expression in all cells and found comparable levels in macrophages and neutrophils, followed by eosinophils, monocytes and T-lymphocytes, and low abundance in B- lymphocytes (Figure 1).

### Lal-/- mice show increased neutral lipid content in peripheral blood and peritoneal lavage cells

**Figure 2:**
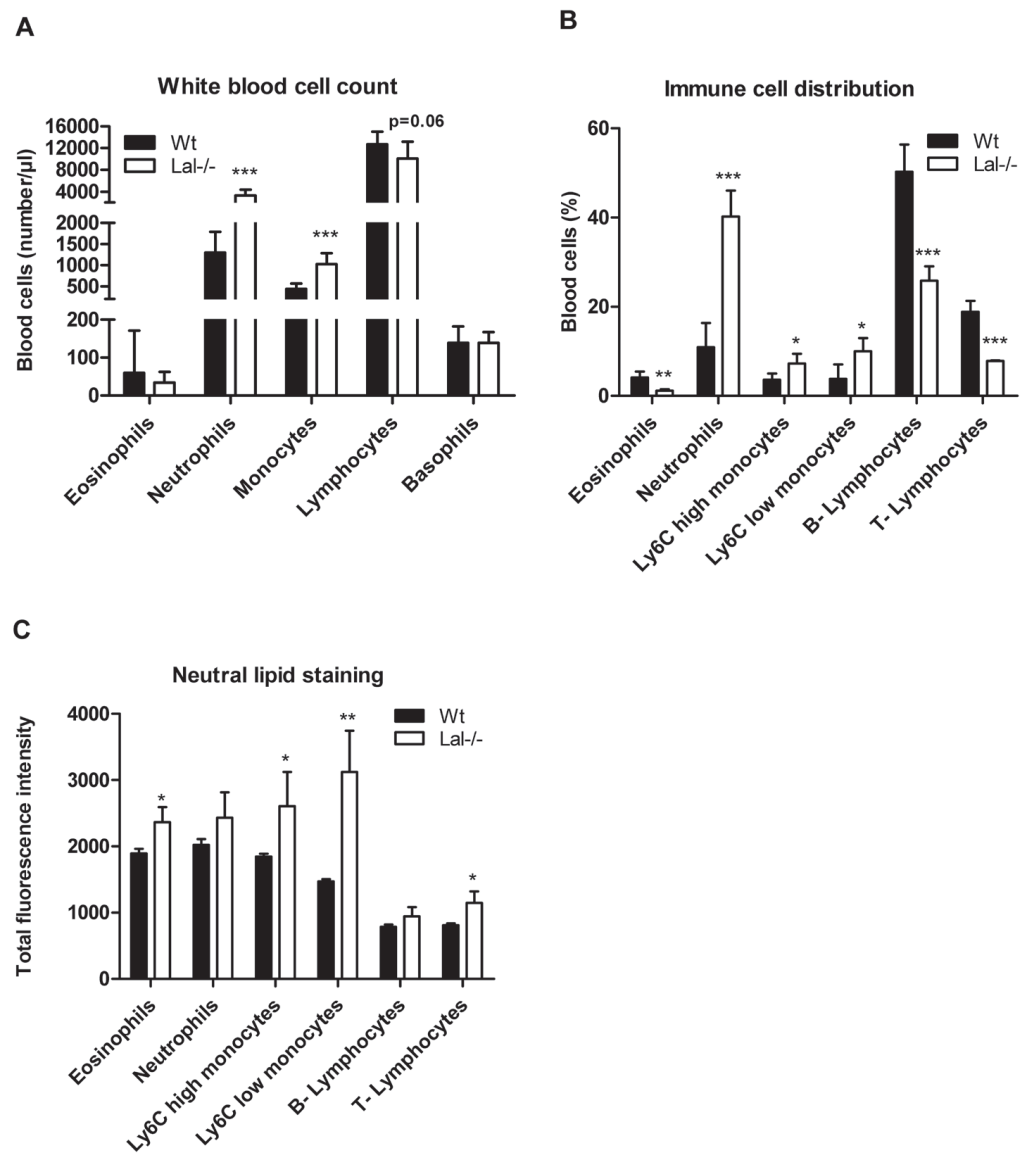
Altered absolute and relative distribution of peripheral blood cells and lipid accumulation in Lal-/- mice **A**. Absolute white blood cell counts as means (*n* = 6-7) + SD. **B**. Immunophenotyping of peripheral blood cells from Wt and Lal-/- mice was performed by flow cytometry. **C**. Neutral lipids in peripheral blood cells were quantified by flow cytometry using BODIPY493/503 as neutral lipid stain. Data in (B, C) represent means of fluorescence intensity + SD (*n* = 5). *p < 0.05; **p ≤ 0.01; ***p ≤ 0.001.

**Figure 3:**
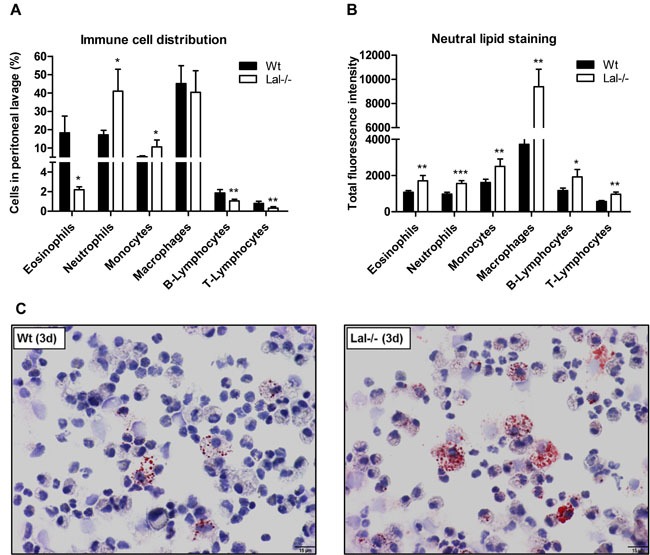
Altered cellular composition and lipid content in peritoneal lavages from Lal-/- mice under inflammatory conditions **A**., **B**. Peritoneal lavage from Wt and Lal-/- mice was collected three days after thioglycolate injection and immunophenotyped using BODIPY493/503 as neutral lipid stain. Data are shown as geometric means of fluorescence intensity + SD (*n* = 5). *p < 0.05; **p ≤ 0.01; ***p ≤ 0.001. **C**. Intracellular neutral lipids of Wt and Lal-/- peritoneal cells were visualized by oil red O/hematoxylin staining. Scale bar: 15 μm.

In accordance with a previous study in myeloid cells [17], immunophenotyping of peripheral blood cells from Wt and Lal-/- mice revealed substantial differences in the blood cell composition with a high increase in absolute numbers and relative abundance of neutrophils and monocytes (Figure 2A, 2B). In addition, we found unchanged absolute numbers of lymphocytes and eosinophils in Lal-/- compared to Wt mice (Figure 2A). Various white blood cells showed an increased amount of neutral lipids in Lal-/- mice (Figure 2C) when using BODIPY as a neutral lipid dye. Among myeloid cells, such as eosinophils and monocytes, particularly monocyte subsets with a low expression profile of Ly6C (Ly6C low) showed the most significant increased amount of neutral lipids in Lal-/- mice. BODIPY staining in lymphocytes revealed significantly increased levels in T-cells but not in B-cells. We next investigated leukocytes in Lal-/- mice during an acute inflammatory response triggered by thioglycolate. We observed (similar as in peripheral blood) an increased relative abundance of neutrophils and monocytes in peritoneal lavage fluid with a concomitant relative reduction of eosinophils and lymphocytes (Figure 3A). Intracellular lipid stainings revealed increased neutral lipid content in all immune cells investigated (Figure 3B). Although the relative abundance of macrophages was comparable between Wt and Lal-/- peritoneal lavages (Figure 3A), macrophages exhibited the highest increase in BODIPY staining (2.5-fold), whereas all other immune cells showed ~1.6-fold increased lipid staining (Figure 3B). Oil red O staining of peritoneal cells collected 3 days after thioglycolate stimulation confirmed neutral lipid accumulation in Lal-/- cells (Figure 3C).

These findings phenocopy and extend the immune phenotypical analyses from peripheral blood showing that neutral lipid accumulation in various cells is even more pronounced under inflammatory conditions.

### Lysosomal accumulation of neutral lipids in Lal-/- macrophages

**Figure 4:**
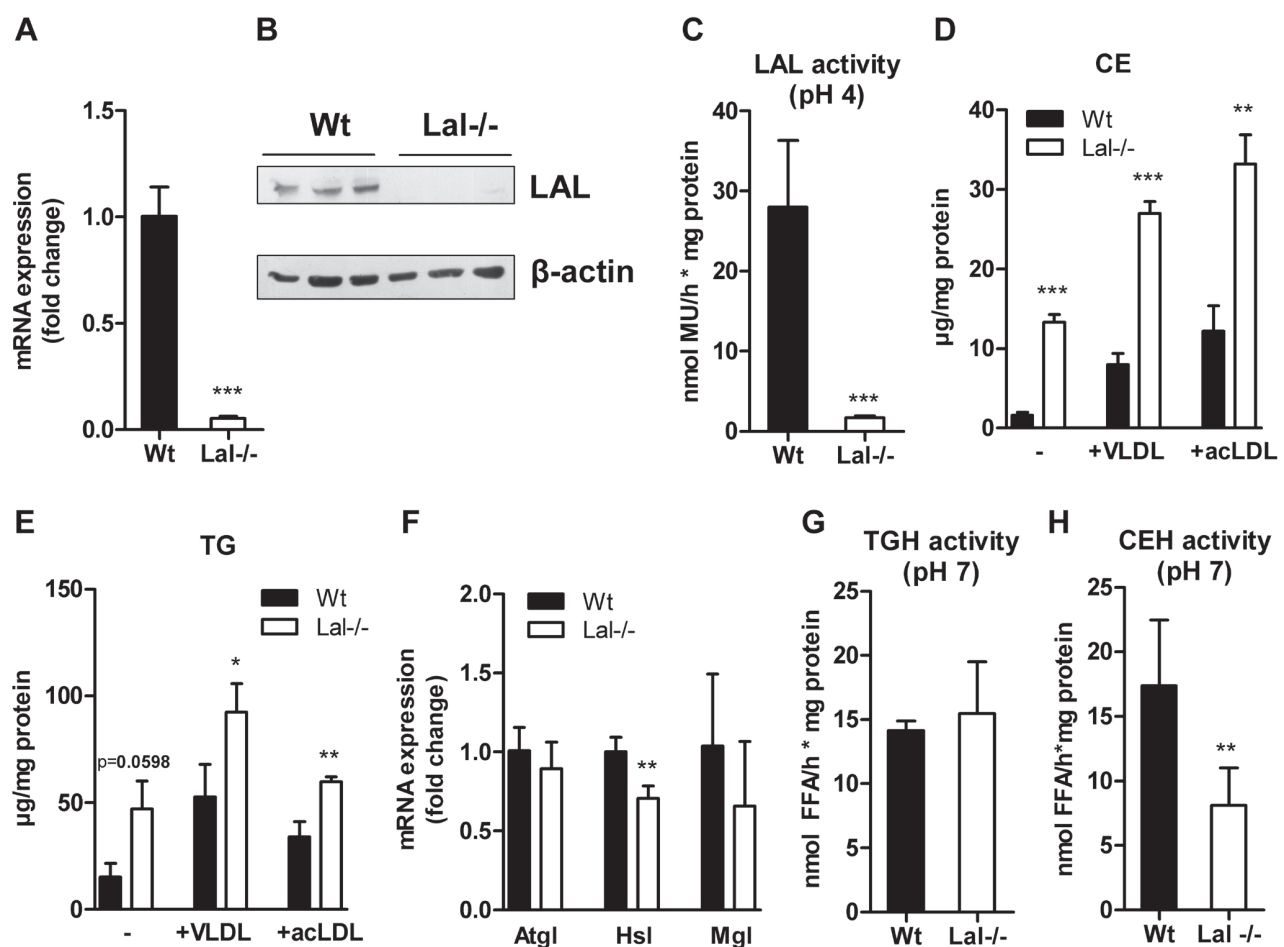
Neutral lipid accumulation in Lal-/- macrophages **A**. mRNA and **B**. protein expression of LAL in Wt and Lal-/- macrophages, normalized to the expression of **A**. cyclophilin and **B**. β-actin. **C**. Acid hydrolase (LAL) activity at pH 4 in macrophage lysates. Biochemical estimation of **D**. CE and **E**. TG concentrations. **F**. Quantitative real-time PCR analysis of Atgl, Hsl, and Mgl mRNA expression. Data represent means (*n* = 4) + SD. **G**. Neutral TG hydrolase and **H**. CE hydrolase activity in macrophage lysates (*n* = 4) + SD. *p < 0.05; **p ≤ 0.01; ***p ≤ 0.001.

Given the observation of high accumulation of neutral lipids in the absence of LAL, we used macrophages as a model to investigate functional consequences of LAL deficiency. To confirm LAL deficiency in macrophages, we performed real time PCR (Figure 4A) and Western blotting analysis (Figure 4B) and measured LAL activity (Figure 4C). Our results demonstrate a complete loss of LAL expression and hydrolytic activity in Lal-/- cells. Consequently, Lal-/- macrophages accumulated CE in basal as well as VLDL- and acLDL-loaded conditions (Figure 4D). TG concentrations in Lal-/- macrophages were higher compared to Wt macrophages, though this effect did not reach statistical significance under basal conditions (Figure 4E). Next, we analyzed whether cytosolic lipid droplets are affected by LAL-D by measuring mRNA expression of neutral lipases and CE and TG hydrolase activities. As shown in Figure 4F, we observed comparable Atgl and monoglyceride lipase (Mgl) mRNA and a decreased hormone-sensitive lipase (Hsl) mRNA expression in Lal-/- *versus* Wt macrophages. Neutral TG hydrolase activities were comparable between Wt and Lal-/- macrophages (Figure 4G). In contrast, CE hydrolase activity at neutral pH was reduced by 53% in Lal-/- cells (Figure 4H).

**Figure 5:**
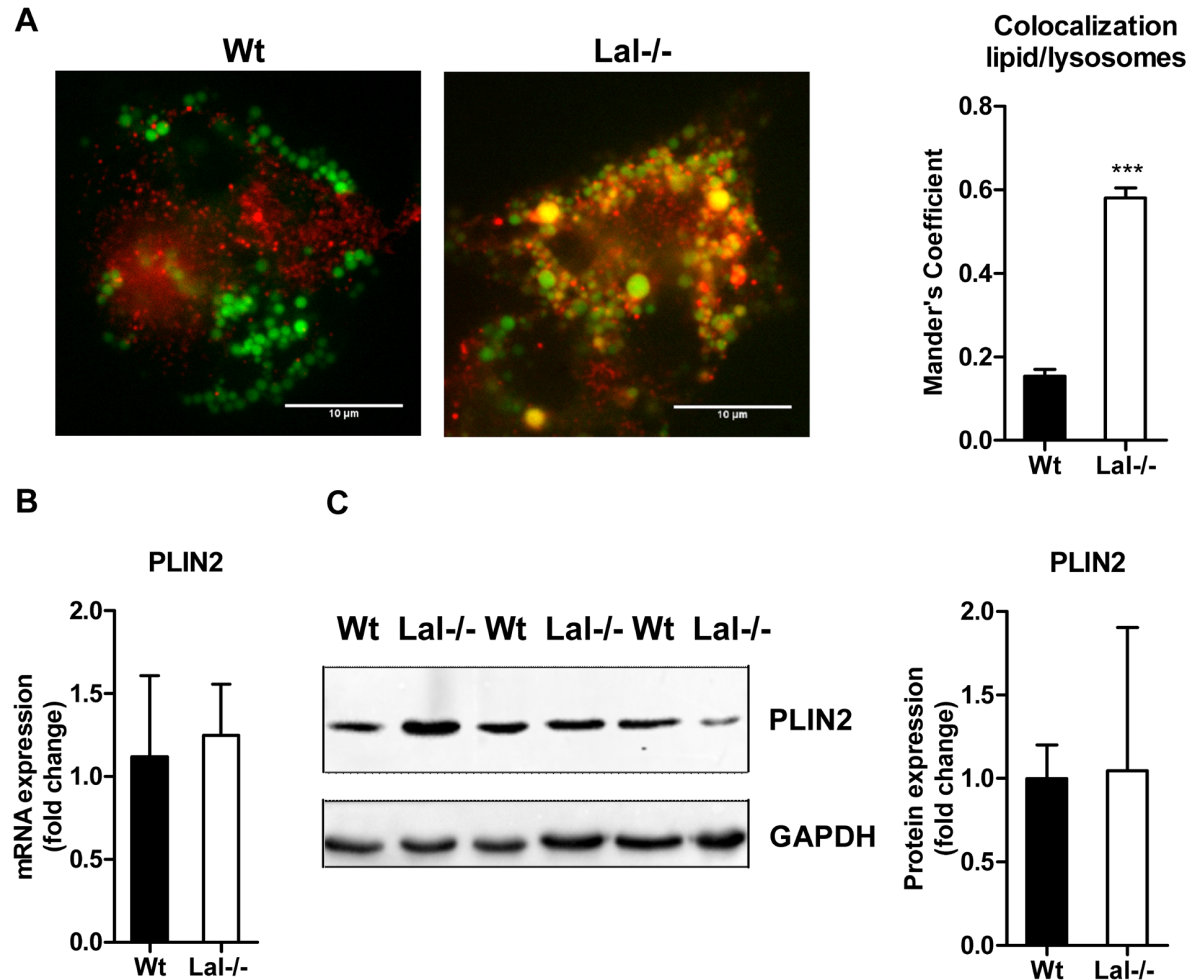
Lysosomal accumulation of lipids in Lal-/- macrophages **A**. Immunofluorescence staining of Wt and Lal-/- macrophages using BODIPY493/503 as neutral lipid dye and Cathepsin D as lysosomal marker. Co-localization analysis was performed using Mander's coefficient (*n* = 4-5) + SEM. **B**. mRNA and **C**. protein expression of the LD marker PLIN2, normalized to the expression of **B**. cyclophilin and **C**. GAPDH. Data are shown as means + SD (*n* = 3-4). ***p ≤ 0.001.

Immunofluorescence staining with BODIPY and Cathepsin D as neutral lipid dye and lysosomal marker, respectively, showed a 3-fold increased lipid colocalization with lysosomes of Lal-/- compared to Wt macrophages (Figure 5A). mRNA and protein expression of the lipid droplet marker perilipin 2 (Plin2) were unaffected (Figure 5B, 5C).

In summary, these data suggest that LAL deficiency in macrophages is accompanied by reduced Hsl expression and neutral CE hydrolase activity, most probably due to an entrapment of CE within the lysosomes and reduced CE availability in cytosolic lipid droplets. Unaltered numbers of cytoplasmic lipid droplets reveal that LAL deficiency does not drastically impact TG trafficking in macrophages under basal conditions.

### Accumulation of 18:1, 18:2, and 20:4 in CE fraction of Lal-/- macrophages

**Figure 6:**
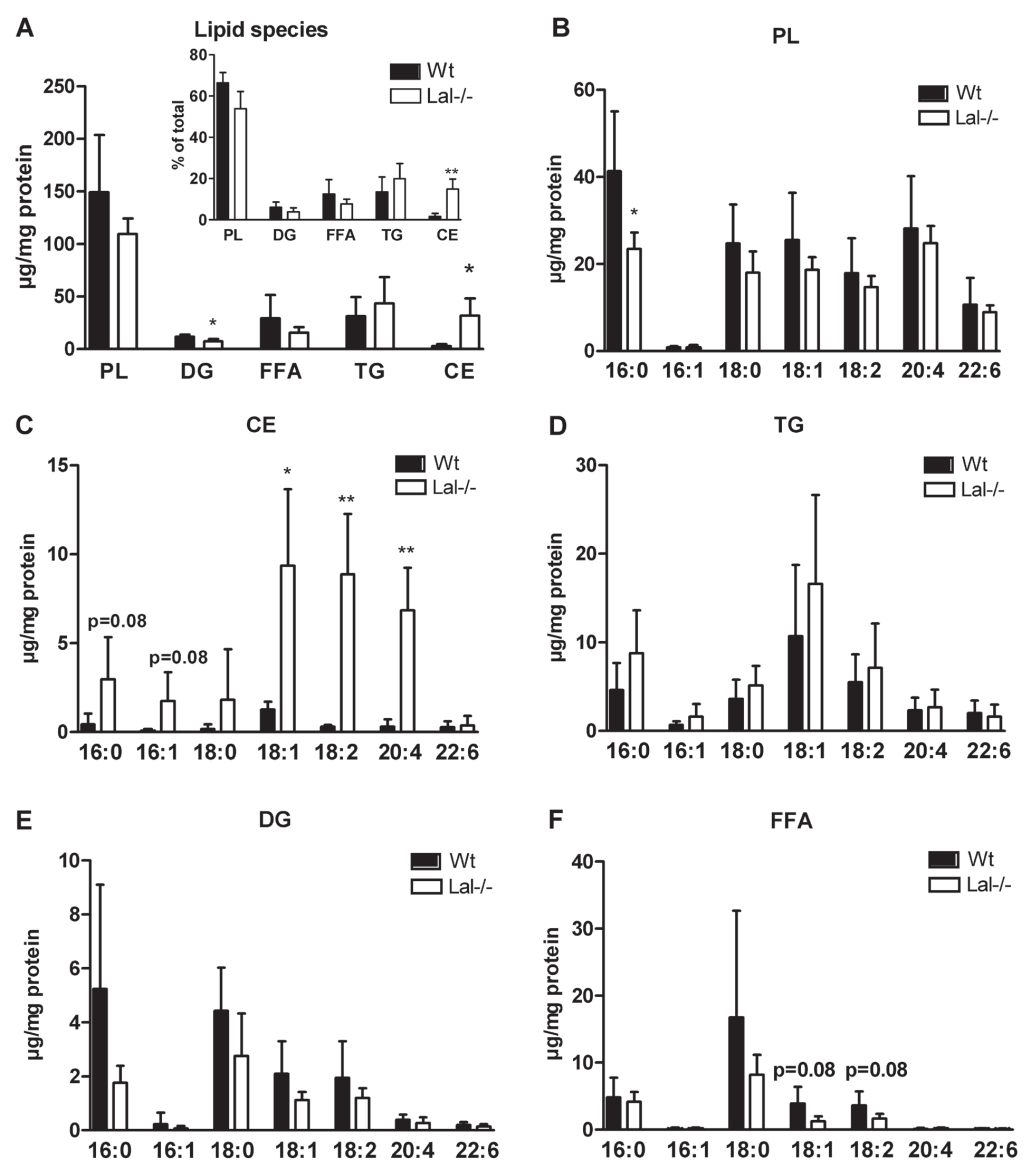
Accumulation of 18:1, 18:2, and 20:4 in CE fraction of Lal-/- macrophages **A**. GC analysis of lipid species in Wt and Lal-/- macrophages. Inset: Percentage distribution of lipid species. **B**.-**F**. GC analysis of FA composition in PL-, CE-, TG-, DG-, and FFA-corresponding bands after TLC separation. Data are shown as mean + SD (*n* = 5). *p < 0.05, **p ≤ 0.01.

Next, we determined the phospholipid (PL) and neutral lipid composition of Lal-/- macrophages by GC. In agreement with observations in Figure 4D and 4E, we found an increase in CE concentrations and slightly decreased diacylglycerol (DG) levels, whereas all other lipid species were unaffected (Figure 6A). Analysis of the relative distribution of the respective lipid classes revealed increased FAs in the CE fraction (inset, Figure 6A). FA composition of PL was comparable between both genotypes with only 16:0 being reduced in Lal-/- macrophages (Figure 6B). In contrast, the FA composition in the CE fraction was markedly different between macrophages of the two genotypes as all FAs analyzed were increased in the CE fraction of Lal-/- cells with most pronounced changes in 16:0, 16:1, 18:1, 18:2, and 20:4 (Figure 6C). The alterations in TG (Figure 6D), DG (Figure 6E), and the non-esterified FA (Figure 6F) compositions were not significantly different. In summary, these data indicate that ineffective CE hydrolysis at acidic pH causes accumulation of FAs with markedly increased levels of 18:2 and 20:4, which are the most important precursors for lipid mediator generation. Therefore, we next investigated whether lipid mediator release is affected by LAL inhibition.

### LAL inhibition in macrophages reduces lipid mediator release

**Figure 7:**
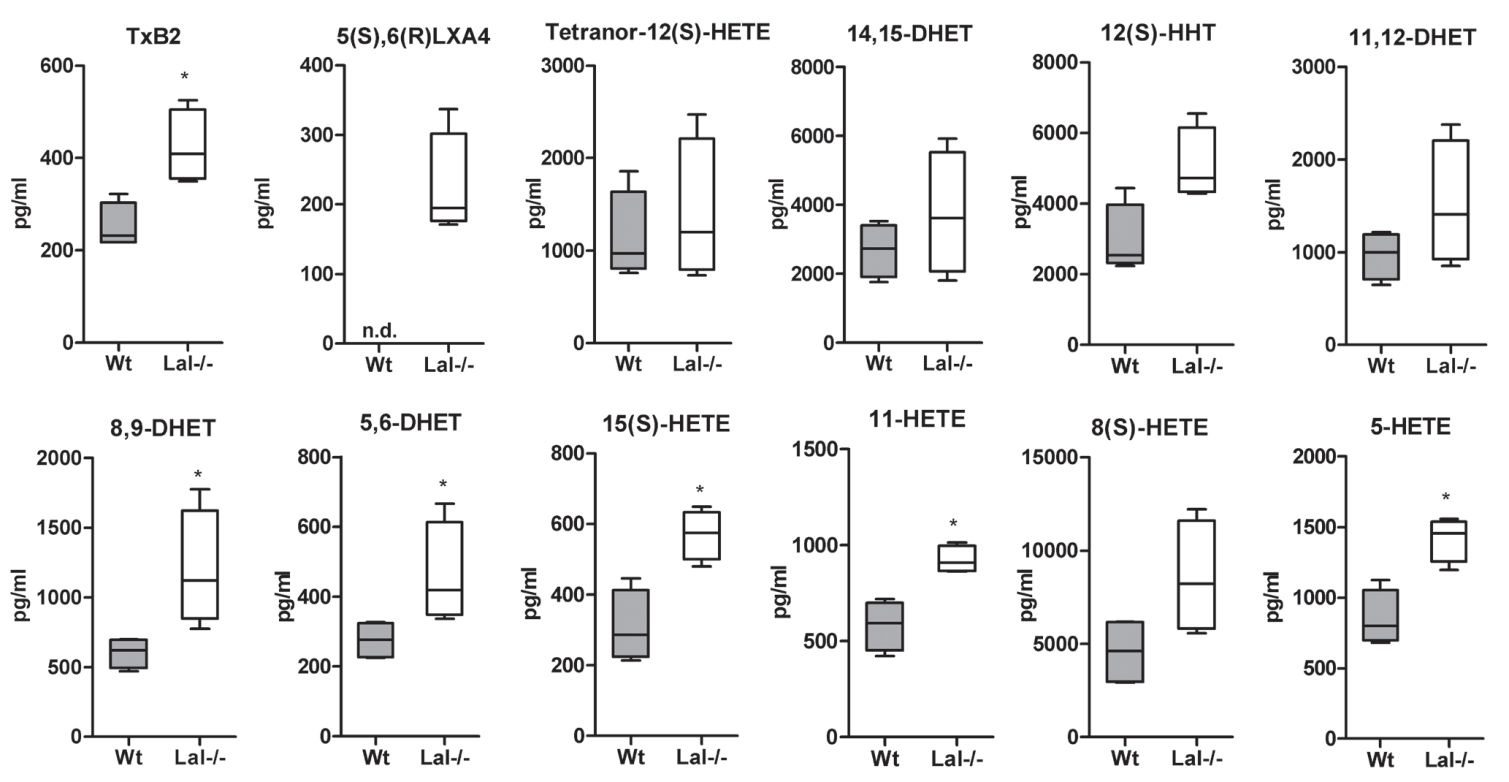
Increased eicosanoid concentrations in plasma of Lal-/- mice Lipid mediator analysis was performed in plasma of Wt and Lal-/- mice by LC-MS/MS analysis. Data are presented as median and range (*n* = 4). n.d., not detectable. *p < 0.05.

To investigate a potential role of lysosomal lipolysis in lipid mediator generation we first measured lipid mediators in the plasma of Wt and Lal-/- mice. Using LC-MS/MS analysis we found that out of 23 lipid mediators detectable in plasma, six 20:4-derived metabolites including TXB2, (5,6)-DHET, (8,9)-DHET as well as 5-, 11-, 15(S)-HETE were increased in Lal-/- mice (Figure 7). This increase may be provoked by the severe phenotype of global LAL deficiency including a massive expansion of myeloid cells in peripheral blood (systemic expansion) and in various tissues (e.g. liver, lung, intestine), where they contribute to local pathogenesis such as tissue injury and inflammation [18].

**Figure 8:**
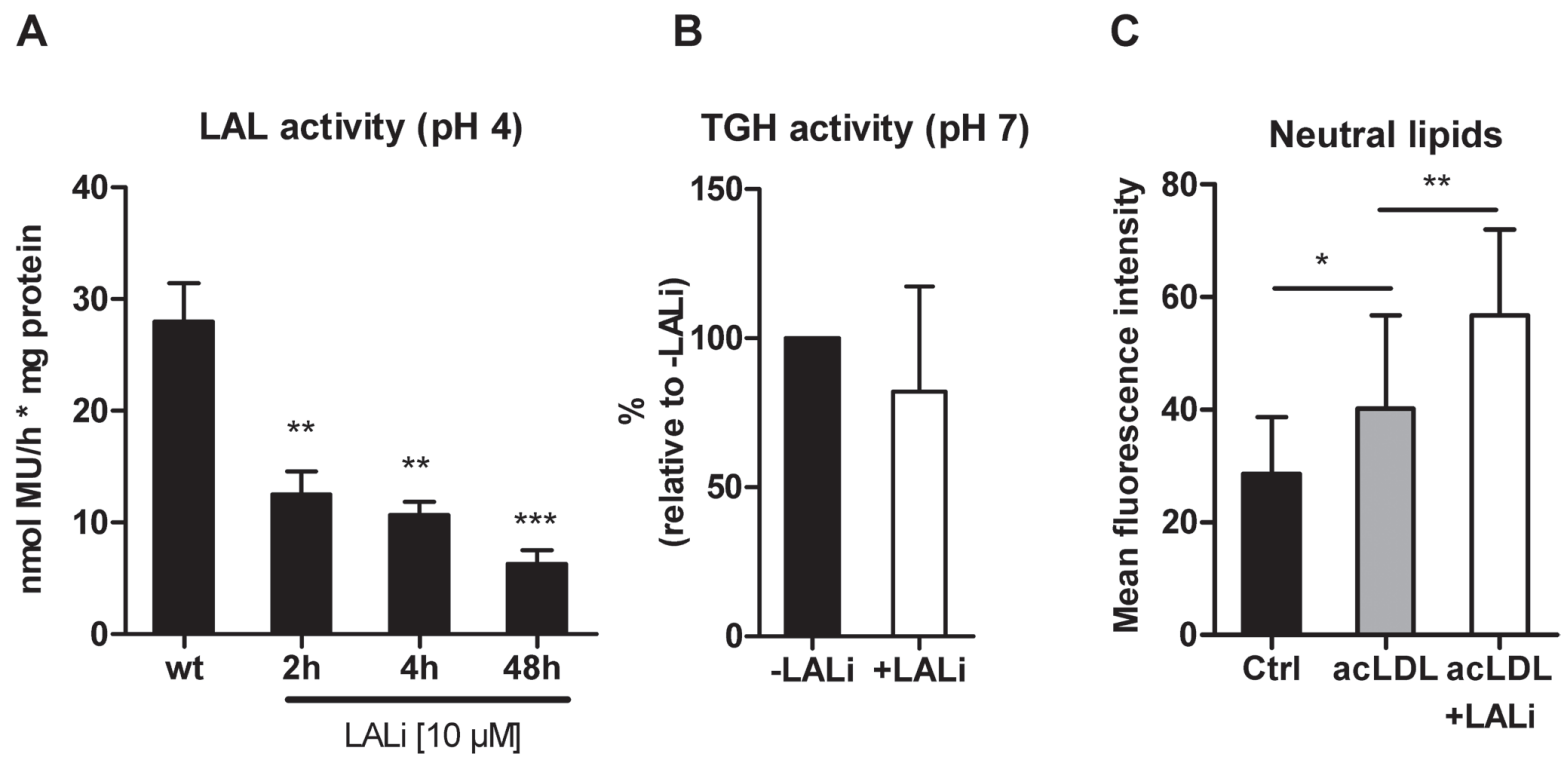
Pharmacological inhibition with LAListat-2 effectively inhibits LAL activity **A**. Wt macrophages were treated with LAListat-2 (10 μM) for 2, 4, and 48 h and LAL activity was determined. Data are presented as means (*n* = 6) + SD. **B**. Wt macrophages were treated with LAListat-2 (10 μM) for 24 h and neutral TGH activity was determined. Data are presented as means (*n* = 9) + SD. **C**. Lipid accumulation in Wt macrophages (Ctrl) and after incubation with acLDL (100 μg/ml, 22 h) in the absence and presence of LAListat-2 (10 μM, 2 h preincubation) was measured by flow cytometry using BODIPY493/503 as neutral lipid stain. Data are shown as geometric means of fluorescence intensity + SD (*n* = 5). *p < 0.05; **p ≤ 0.01; ***p ≤ 0.001.

To circumvent these alterations, we used an alternative strategy and inhibited LAL in Wt macrophages by treatment with the pharmacological inhibitor LAListat-2. For this purpose, we first treated Wt macrophages with LAListat-2 for 2, 4, and 48 h, which markedly reduced LAL activity up to 78% (Figure 8A). To test the specificity of LAListat-2 we determined neutral TG hydrolase activity, which remained unaffected in response to inhibitor treatment for 24 h (Figure 8B). In addition, when loaded with acLDL in the absence and presence of LAListat-2 for 22 h, Wt macrophages showed an increased intracellular lipid content, as quantified by flow cytometry using BODIPY (Figure 8C). Together, these findings support the conclusion that the inhibitor specifically blocks LAL-mediated lipolysis, which consequently results in neutral lipid accumulation.

**Figure 9:**
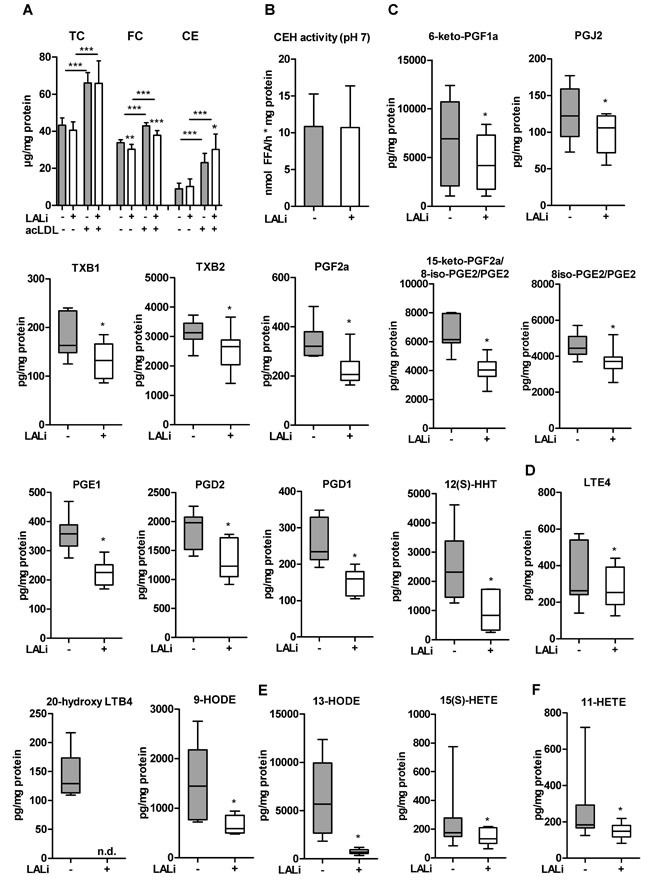
LAListat-2 reduces lipid mediator release in peritoneal macrophages Thioglycolate-elicited macrophages were loaded with acLDL (100 μg protein/ml, 6 h) in the absence or presence of the pharmacological LAL inhibitor LAListat-2 (10 μM, 6 h). **A**. Cholesterol concentrations, **B**. neutral CE hydrolase activity, and lipid mediators derived from **C**. COX, **D**. LOX, **E**. LOX/CYP, and **F**. COX/CYP pathways. Lipid mediator concentrations were determined by LC-MS/MS analysis. Data are presented as (A, B) means (*n* = 7-9) + SD and **E**.-**F**. median and range (*n* = 6). n.d., not detectable. *p < 0.05.

Of particular interest was to study the release of lipid mediators from Wt macrophages after LAListat-2-mediated LAL inhibition. To ensure the availability of LAL substrate and to trigger LAL activity we incubated thioglycolate-elicited macrophages with acLDL for 6 h in the absence and presence of LAListat-2, resulting in comparable uptake of acLDL, decreased FC, and increased intracellular CE concentrations (Figure 9A). LAListat-2 treatment did not affect neutral CE hydrolase activity (Figure 9B). We observed reduced release of lipid mediators from all three major enzymatic pathways: the cyclooxygenase (COX), the lipoxygenase (LOX) and the cytochrome P450 (CYP) pathways (Figure 9C-9F). Reduced metabolite levels included those of the COX-derived 6-keto-PGF_1α_, PGJ_2_, TXB_1_, TXB_2_, PGF_2α_, 15-keto-PGF_2α_, PGE_2_, PGE_1_, PGD_2_, PGD_1_, and 12(S)-HHT (Figure 9C), the LOX-derived 20-hydroxy LTE_4_, LTB_4_, and 9-HODE (Figure 9D), the LOX/CYP-derived 13-HODE and 15(S)-HETE (Figure 9E), and the COX/CYP-derived 11-HETE (Figure 9F). The effect of LAL inhibition was not restricted to eicosanoids arising from 20:4 as we also observed significantly reduced levels of 18:2- and 20:3-derived metabolites (9-HODE, 13-HODE, and PGD_1_, respectively. Since the uptake of acLDL was not compromised by LAL inhibition (Figure 9A), these findings demonstrate that LAL inhibition in macrophages results in a reduced release of lipid mediators. Unchanged neutral CE hydrolase activity (Figure 9B) demonstrates that lysosome-derived lipids serve as precursors for lipid mediator generation in these cells.

## DISCUSSION

Beyond processing cellular waste, lysosomes are emerging as functional organelles that can sense nutrient availability (reviewed in [19]) and control and maintain cellular energy homeostasis. By mobilizing FAs from lipoproteins, lysosomes generate lipid signaling molecules which may either directly (through binding to PPARs) or indirectly (through their derivatives and metabolites) regulate cellular function. Bioactive lipid metabolites, such as eicosanoids, lysophosphatidic acid, and ceramides affect immune cell biology and inflammatory responses [20]. Receptor-mediated uptake and lysosomal hydrolysis of endocytosed LDL was shown earlier to provide substrates for prostaglandin and leukotriene synthesis in immune cells [21]. In human blood-derived monocytes, LDL-derived 20:4 was shown to be utilized for the production of PGI_2_, TXA_2_, PGE_2_, LTC_4_, and LTB_4_ [21]. Whether 20:4 is directly provided from lipoproteins or indirectly from downstream metabolic processes as well as the enzymatic machinery to release these FAs remained elusive.

Studies using Lal-/- mice have provided substantial evidence for a significant role of LAL in the regulation of inflammation-relevant functions in various tissues. A major phenotype of LAL-D in mice is the abnormal appearance of macrophages within multiple organs, predominantly in lung, intestine, liver, and spleen, resulting in inflammation and hepatosplenomegaly [8, 18]. Lal-/- mice suffer from an expansion of CD11b+, Gr-1+ cell lineage (myeloid-derived suppressor cells) and show highly reduced numbers of peripheral lymphocytes, thus implicating LAL as a critical factor for lymphoid and myeloid progenitor cell development and function [17]. Our observations further extend these studies showing that not only macrophages, but also other peripheral blood cells from Lal-/- mice accumulate neutral lipids. Although lymphocytes express Lal mRNA, they are not professional phagocytes and it is unexpected that lymphocytes accumulate lipids derived from the endocytic pathway. In fact, based on the methods used here, we cannot discriminate whether Lal-/- lymphocytes have an increased lipid content in lipid droplets or lysosomes. The observation of an increased presence of cytokines [10, 18, 22] and lipid mediators in Lal-/- mice together with the fact that these inflammatory mediators are known triggers for lipid droplet generation [23] allow to speculate that rather secondary effects rather than loss of LAL in lymphocytes itself might cause this increase. Given the scarce literature regarding lipid droplets in lymphoid cells, this finding is, however, highly interesting and requires further studies.

In the absence and presence of lipoproteins, Lal-/- macrophages are characterized by a moderate increase in intracellular TG levels but a substantial elevation of esterified cholesterol concentrations, comparable with recent observations in the liver of Lal-/- mice [5, 24]. Immunofluorescence co-staining with the neutral lipid dye BODIPY and Cathepsin D as lysosomal marker revealed a 3-fold increase in co-localization of lipids with lysosomes in Lal-/- compared to Wt macrophages, indicating that neutral lipids are mainly entrapped within the lysosome. Although LAL activity is completely absent, neutral TG hydrolase activity remains fully functional and lipid droplet abundance (verified by Plin2 expression) unchanged. These observations indicate unaltered ability of neutral TG mobilization in Lal-/- macrophages. In contrast, decreased Hsl mRNA expression and reduced neutral CE hydrolase activity imply reduced neutral CE hydrolysis in Lal-/- macrophages. Since the number of lipid droplets is unaffected by LAL deficiency, and Hsl -/- macrophages do not suffer from CE accumulation [25] it is unlikely that neutral CE hydrolase activity accounts for the changes in CE-derived FAs. Together, our results reveal an important role for LAL-driven lysosomal lipid degradation, which seems to be an active process predominantly in myeloid cells causing lipid accumulation mainly in the form of CE.

FA analysis of the main lipid classes revealed 18:2- and 20:4 as most abundant FAs accumulating in the CE fraction of Lal-/- macrophages. It was therefore surprising that eicosanoid concentrations in plasma were increased by LAL-D. In this context, it is important to note that systemic LAL-D in mice causes a severe phenotype including elevated concentrations of transaminases, indicating liver damage and inflammation, systemically increased pro-inflammatory cytokine and leukocyte profiles, and macrophage accumulations in multiple organs [8, 10, 24]. Apart from TXB_2_, which is a COX-derived mediator, all other mediators that were altered in Lal-/- plasma originated from the CYP-pathway. CYP-derived arachidonic acid metabolites represent a group of mediators that participate in the regulation of liver metabolic activity and hemodynamics [26]. The fact that Lal-/- mice develop hepatomegaly and progressive liver disease with hyperlipidemia, Kupffer cell infiltration, and inflammation may explain the observed increase in plasma eicosanoid concentrations. Our observations are in line with previous reports showing that LAL-D affects immune homeostasis due to both cell-intrinsic and microenvironmental effects [9, 17]. Thus, systemic inflammation in Lal-/- mice may cause a cumulative effect and could account for the observed increase in eicosanoids in the plasma of Lal-/- mice.

To circumvent the potential systemic effects of chronic LAL-D, we treated Wt cells *ex vivo* with LAListat-2 and measured lipid mediator release from these cells. As a consequence of reduced LAL-mediated lipid degradation, we observed a decreased release of 18:2- and 20:4-derived lipid mediators from macrophages after acute LAL inhibition. Therefore, we demonstrate a role of LAL-mediated lysosomal degradation in the generation of precursors for lipid mediator synthesis. In agreement with earlier studies [27–29], we confirmed LAListat-2 as a specific inhibitor of LAL. In fact, the reduced release of a number of lipid mediators derived from all three enzymatic pathways can be attributed to LAL inhibition. It is known that lipid mediator generation is regulated in a highly time-dependent manner at different levels, e.g. through the availability of enzymes, substrates, and the sites of mediator generation within different cellular compartments [14]. Whether lysosomal lipolysis provides substrates directly for eicosanoid generation or indirectly by replenishing FA in other subcellular pools for the subsequent release remains to be determined. The observed pan-reduction of lipid mediators caused by LAL inhibition argues for the latter option.

In summary, this study provides mechanistic evidence that LAL-driven lipolysis provides a source for lipid mediator precursors and suggests a functional role of lysosomes as signaling organelles in immune cells.

## MATERIALS AND METHODS

### Animals

Lal-/- mice [8] were backcrossed on a C57BL/6 background [24]. Sex-and age-matched (12-16 weeks) Lal-/- and Wt littermates were maintained in a clean environment with unlimited access to chow and water in a regular light-dark cycle (12 h/12 h). Animal experiments were carried out in accordance with the EU Directive 2010/63/EU and approved by the Federal Ministry of Science, Research, and Economy, Vienna, Austria.

### Isolation of inflammatory cells for quantitative real-time PCR

Cells were flow-sorted using a BD FACSAria III Cellsorter (BD Biosciences, San Jose, CA). Peritoneal lavages from Wt mice two days after thioglycolate injection were used to sort eosinophils, neutrophils, monocytes, and macrophages. B- and T-lymphocytes were sorted from peripheral blood after red blood cell lysis in ammonium-chloride-potassium (ACK) lysis buffer (150 mM NH_4_Cl, 10 mM KHCO_3_, 0.1 mM Na_2_EDTA, pH 7.2). Total RNA from cells was extracted using TriFast reagent according to the manufacturer's protocol (Peqlab, Erlangen, Germany). One μg of total RNA was reverse transcribed using the High Capacity cDNA Reverse Transcription Kit (Applied Biosystems, Carlsbad, CA). Quantitative real-time PCR was performed on a LightCycler 480 (Roche Diagnostics, Rotkreuz, Switzerland) using the QuantifastTM SYBR^®^ Green PCR kit (Qiagen, Hilden, Germany). For data normalization, Gapdh was used as an endogenous control and the relative units for gene expression were calculated by using the 2^-ΔΔct^ method. The following primer sequences were used: Cyclophilin-fw 5’-CATCCAGCCATTCAGTCTT-3’, Cyclophilin-rv 5’-TTCCAGGATTCATGTGCCAG-3’; Gapdh-fw 5′-AGGTCGGTGTGAACGGATTTG-3′, Gapdh-rv 5′-GGGGTCGTTGATGGCAACA-3′; Atgl-fw 5′-GCCACTCACATCTACGGAGC-3′, Atgl-rv 5′-GACAGCCACGGATGGTGTTC-3′; Hsl-fw 5′-GCTGGTGACACTCGCAGAAG-3′, Hsl-rv 5′-TGGCTGGTGTCTCTGTGTCC-3′; Lal-fw 5’-GCAAAGGTCCCAGACCAGTT-3’, Lal-rv 5’-TCATCAAAACTGAAGGCCCAGA-3’; Mgl-fw 5′-CGGACTTCCAAGTTTTTGTCAGA-3′, Mgl-rv 5′-GCAGCCACTAGGATGGAGATG-3′; Plin2-fw 5′-GACCTTGTGTCCTCCGCTTAT-3′, Plin2-rv 5′-CAACCGCAATTTGTGGCTC-3′.

### Isolation and cultivation of peritoneal macrophages

Peritoneal macrophages were collected 72 h after an intraperitoneal injection of 2.5 ml 3% thioglycolate broth as described [30]. To establish the inhibitory action of the LAL inhibitor LAListat-2, macrophages cultured in DMEM/10% LPDS were incubated with 10 μM LAListat-2 for 2, 4, and 48 h. For flow cytometric analyses, macrophages were preincubated with LAListat-2 (10 μM, 2 h) and then loaded for 22 h with acetylated (ac)LDL (100 μg protein/ml medium). To estimate lipoprotein uptake, cells were incubated with acLDL in the presence or absence of Lalistat-2 for 6 h. To study foam cell formation Wt and Lal-/- macrophages were cultivated for 24 h in the absence or presence of VLDL or acLDL (100 μg protein/ml medium) after which lipid content was measured as previously described [30].

### White blood cell counts

Complete blood cell counts were performed with the Cell Counter Analyzer MS9-5V (Melet Schloesing Laboratories GmbH, Maria Enzersdorf, Austria).

### Immunophenotyping and lipid staining of peripheral blood and peritoneal cells

Peripheral blood cells were collected by retro-bulbar puncture from isoflurane anesthetized mice. Peritoneal lavage cells were collected as mentioned above. After red blood cell lysis, remaining cells were fixed with 10% methanol-free formalin for 10 min at 4°C and blocked with PBS/10% FCS for 10 min at 4°C. For flow cytometric analyses, cells were stained with antibodies as described recently [15]. Briefly, we used CD11b-Alexa Fluor647, SiglecF-PE, Gr-1-APC-Cy7 antibodies (BD Biosciences, San Jose, CA), CD115-PE-Cy7, CD19-eFluor605, CD3-eFluor450 antibodies (eBioscience Inc, San Diego, CA) for peripheral blood cells, and F4/80-eFluor450 (eBioscience), SiglecF-PE, Gr-1-PerCP-Cy5.5, CD19-PE-Cy7 and CD3-APC antibodies (BD Biosciences) for peritoneal cells. Intracellular neutral lipids were quantified by staining with BODIPY493/503 (1 μg/ml; Life Technologies, Carlsbad, CA) for 10 min at 4°C. Cells (1 × 10^5^ cells/measurement) were analyzed using an LSR II flow cytometer (BD Biosciences). Data were acquired using DIVA 6.1.2 software (BD Biosciences) and the analysis was performed using FlowJo (Treestar Inc., San Carlos, CA).

### Oil red O (ORO) stainings

Cells from the peritoneal lavage were sedimented by cytocentrifugation onto glass slides and fixed with 10% methanol-free formalin. Cells were stained with ORO for 30 min and counterstained with Mayer's Hematoxylin. Coverslips were mounted with Aquamount (Dako, Glostrup, Denmark). Microscopic images were taken using a Nikon Eclipse E600 microscope equipped with a Nikon Digital DS-U1 unit (Spach Optics Inc., New York, NY).

### Immunofluorescence microscopy

Immunofluorescence microscopy was performed as described [31] with minor modifications. Briefly, macrophages were plated on coverslips in DMEM/10% LPDS for 24 h. Cells were fixed with 10% methanol-free formalin, washed with PBS and blocked with 1% BSA in PBS containing 0.2 M glycine. Thereafter, cells were incubated with anti-cathepsin D primary antibody (1:1200; Abcam, Cambridge, United Kingdom) in 1% BSA/PBS at 4°C overnight. Macrophages were washed three times with PBS for 5 min and incubated with secondary antibody (1:250, goat anti-mouse IgG_1_ Alexa Fluor^®^594 conjugated; Thermo Fisher Scientific, Waltham, MA) and BODIPY493/503 (1 μg/ml) for 1 h at RT. After washing, slides were mounted in Dako fluorescent mounting medium (Dako Denmark A/S, Glostrup, Denmark). Confocal images were acquired and analyzed as described [31].

### Western blotting analysis

Protein samples (40 μg protein/lane) of lysed macrophages were separated by SDS-PAGE and transferred to nitrocellulose membranes. The blots were incubated with anti-rabbit polyclonal antibodies against LAL (1:500; Seven Hills Bioreagents, Cincinnati, OH) and GAPDH (1:1,000; Cell Signaling Technology, Danvers, MA), an anti-guinea pig polyclonal antibody against PLIN2 (1:1,000; GP40; 1:5,000; Progen, Heidelberg, Germany), anti-rabbit (1:2,500), and a monoclonal anti-mouse β-actin antibody (1:10,000; Sigma, Vienna, Austria). The horseradish peroxidase-conjugated goat anti-rabbit (1:10,000; Pierce, Bonn, Germany), rabbit anti-guinea pig (1:10,000; Southern Biotech, Birmingham, AL), and rabbit anti-mouse antibodies (1:1,000) (Dako, Glostrup, Denmark) were visualized by enhanced chemiluminescence detection (ECL Prime; Amersham Biosciences, Piscataway, NJ) using AGFA Curix Ultra X-Ray films (Siemens, Graz, Austria) or Chemidoc (BioRad).

### Lipase activities

Macrophages were lysed in lysis buffer (100 mM potassium phosphate, 250 mM sucrose, 1 mM EDTA, 1 mM DTT, pH 7), sonicated on ice (2×10 s with 1 min interval), and centrifuged at 1,000 x *g* and 4°C for 10 min. The protein content of the supernatant was determined by a Lowry assay (Bio-Rad Laboratories, Hercules, CA). Fifty μg of protein from macrophage lysates were diluted to a final volume of 100 μl with lysis buffer. To measure neutral TG hydrolase activity, the samples were mixed with 100 μl of TG substrate [0.3 mmol triolein/sample, 0.5 μCi/sample [9, 10 3H(N)]triolein (Perkin Elmer, Waltham, MA), 3.5 μg mixed micelles of phosphatidylcholine and phosphatidylinositol (3:1, w:w)]. Neutral CE hydrolase activity was determined in samples mixed with CE substrate [0.2 mmol cholesteryl oleate/sample, 0.04 μCi/sample cholesteryl [1-^14^C]oleate (Amersham Biosciences, Piscataway, NJ), 35.5 μg mixed micelles of phosphatidylcholine and phosphatidylinositol (3:1, w:w)]. Each substrate contained FFA-free BSA at a final concentration of 2% in 100 mM phosphate buffer. After incubation in a water bath for 1 h at 37°C, the reaction was stopped by the addition of 3.25 ml stop solution (methanol:chloroform:n-heptane, 10:9:7, v:v:v) and 1 ml of 0.1 M potassium carbonate (pH 10.5, adjusted with boric acid). The tubes were vortexed for 10-15 s and centrifuged at 1,700 x *g* for 15 min at 4°C. The radioactivity in 1 ml of the upper phase was determined by liquid scintillation counting, and the release of FAs was calculated.

LAL activity was estimated using the fluorogenic substrate 4-methyl-umbelliferyl-palmitate (4-MUP) as described [32] with some modifications. Mouse peritoneal macrophages were washed twice with ice cold PBS and scraped in lysis buffer (100 mM KH_2_PO_4_, pH 6.8, 1 mmol/l ethylenediamine tetraacetic acid, 10 mmol/l dithiothreitol, 0.5% NP-40, 0.02% sodium azid, protease inhibitors). Protein concentrations were quantified by a Lowry assay. Per sample, 2 μl of 4-MUP (10 mM) were mixed with 2 μl phosphatidylcholine (16 mM) in hexane, evaporated under a stream of nitrogen, and resuspended in 50 μl sodium taurocholate (2.4 mM) by sonication. Fifty microliters of 4-MUP substrate, 125 μl assay buffer (200 mM sodium acetate pH 4.5), and 25 μl cell lysate were incubated for 30 min at 37°C. The reaction was stopped by the addition of 100 μl of 0.75 M Tris (pH 11). Relative fluorescence units (RFU) were determined at 360 nm excitation/460 nm emission on a Victor 1420 multilabel counter (PerkinElmer, Waltham, MA) using 4-MU as standard. LAL activity is expressed as nmol MU/h*mg protein.

### Quantitation of lipid mediators

Peritoneal macrophages were cultivated in DMEM/10% LPDS containing acLDL (100 μg/ml) in the absence or presence of 10 μM LAListat-2 for 6 h. Thereafter, the medium was replaced by HBSS containing Ca^2+^ and Mg^2+^. After 1 h of incubation, the supernatants were collected and centrifuged at 400 x *g* for 10 min at 4°C. Supernatants were immediately frozen in liquid N_2_ and stored at -80°C until analysis. LC-MS/MS analysis of 200 μl cell supernatant and plasma from Wt and Lal-/- mice was performed on a 5500 QTrap mass spectrometer (AB Sciex, Darmstadt, Germany) operating in negative electrospray ionization mode [33].

### FA composition by GC analysis

Lipids were extracted from macrophages twice with hexane:isopropanol (3:2, v:v), dried under a stream of nitrogen, and redissolved in 1 ml of chloroform. Aliquots of 700 μl were separated by TLC under argon using hexane:diethylether:acetic acid (70:30:1; v:v:v) as mobile phase. Plates were dried, standards were stained with iodine vapor, and PL-, DG-, FFA-, TG-, and CE-corresponding bands were scraped off the plates. After addition of the internal standard (pentadecanoic acid), lipids were transesterified (1 ml toluene and 1 ml boron trifluoride-methanol (20 %) directly on the silica gel at 110°C for 1 h. GC analysis of the corresponding FA methyl esters was performed as described [34]. FA composition was quantitated by peak area comparison with the internal standard and normalized to protein concentrations.

### Statistical analysis

Statistical analyses were performed using GraphPad Prism 5.0 software. Significance was calculated by Student's *t*-test and Welch correction in cases of unequal variances. Data are shown as means + SD. For statistical analysis of lipid mediators, non-parametric Wilcoxon signed-rank tests and Mann-Whitney U tests were used. Data are expressed as median with minimum and maximum range. The following levels of statistical significance were used: *, *p* < 0.05; **, *p* ≤ 0.01; ***, *p* ≤ 0.001.

## Abbreviations

acLDL: acetylated low-density lipoprotein
ATGL: adipose triglyceride lipase
CE: cholesteryl ester
FA: fatty acid
HSL: hormone-sensitive lipase
LAL-D: lysosomal acid lipase deficiency
MGL: monoglyceride lipase
PL: phospholipid
TG: triglyceride

## References

[1] Brown MS, Goldstein JL (1976). Receptor-mediated control of cholesterol metabolism. Science.

[2] Levine B, Kroemer G (2008). Autophagy in the pathogenesis of disease. Cell.

[3] Singh R, Kaushik S, Wang Y, Xiang Y, Novak I, Komatsu M, Tanaka K, Cuervo AM, Czaja MJ (2009). Autophagy regulates lipid metabolism. Nature.

[4] Sheriff S, Du H, Grabowski GA (1995). Characterization of lysosomal acid lipase by site-directed mutagenesis and heterologous expression. J Biol Chem.

[5] Grumet L, Eichmann TO, Taschler U, Zierler KA, Leopold C, Moustafa T, Radovic B, Romauch M, Yan C, Du H, Haemmerle G, Zechner R, Fickert P (2016). Lysosomal Acid Lipase Hydrolyzes Retinyl Ester and Affects Retinoid Turnover. J Biol Chem.

[6] Bernstein DL, Hülkova H, Bialer MG, Desnick RJ (2013). Cholesteryl ester storage disease: review of the findings in 135 reported patients with an underdiagnosed disease. J Hepatol.

[7] Krivit W, Freese D, Chan KW, Kulkarni R (1992). Wolman's disease: a review of treatment with bone marrow transplantation and considerations for the future. Bone Marrow Transplant.

[8] Du H, Heur M, Duanmu M, Grabowski GA, Hui DY, Witte DP, Mishra J (2001). Lysosomal acid lipase-deficient mice: depletion of white and brown fat, severe hepatosplenomegaly, and shortened life span. J Lipid Res.

[9] Qu P, Du H, Wilkes DS, Yan C (2009). Critical roles of lysosomal acid lipase in T cell development and function. Am J Pathol.

[10] Yan C, Lian X, Li Y, Dai Y, White A, Qin Y, Li H, Hume DA, Du H (2006). Macrophage-specific expression of human lysosomal acid lipase corrects inflammation and pathogenic phenotypes in lal-/- mice. Am J Pathol.

[11] Ouimet M, Franklin V, Mak E, Liao X, Tabas I, Marcel YL (2011). Autophagy regulates cholesterol efflux from macrophage foam cells via lysosomal acid lipase. Cell Metab.

[12] Huang SC, Everts B, Ivanova Y, O’Sullivan D, Nascimento M, Smith AM, Beatty W, Love-Gregory L, Lam WY, O’Neill CM, Yan C, Du H, Abumrad NA (2014). Cell-intrinsic lysosomal lipolysis is essential for alternative activation of macrophages. Nat Immunol.

[13] Dennis EA, Norris PC (2015). Eicosanoid storm in infection and inflammation. Nat Rev Immunol.

[14] Bozza PT, Bakker-Abreu I, Navarro-Xavier RA, Bandeira-Melo C (2011). Lipid body function in eicosanoid synthesis: an update. Prostaglandins Leukot Essent Fatty Acids.

[15] Schlager S, Goeritzer M, Jandl K, Frei R, Vujic N, Kolb D, Strohmaier H, Dorow J, Eichmann TO, Rosenberger A, Wölfler A, Lass A, Kershaw EE (2015). Adipose triglyceride lipase acts on neutrophil lipid droplets to regulate substrate availability for lipid mediator synthesis. J Leukoc Biol.

[16] Dichlberger A, Schlager S, Maaninka K, Schneider WJ, Kovanen PT (2014). Adipose triglyceride lipase regulates eicosanoid production in activated human mast cells. J Lipid Res.

[17] Qu P, Shelley WC, Yoder MC, Wu L, Du H, Yan C (2010). Critical roles of lysosomal acid lipase in myelopoiesis. Am J Pathol.

[18] Lian X, Yan C, Yang L, Xu Y, Du H (2004). Lysosomal acid lipase deficiency causes respiratory inflammation and destruction in the lung. Am J Physiol Lung Cell Mol Physiol.

[19] Settembre C, Fraldi A, Medina DL, Ballabio A (2013). Signals from the lysosome: a control centre for cellular clearance and energy metabolism. Nat Rev Mol Cell Biol.

[20] Chen SH, Oyarzabal EA, Hong JS (2016). Critical role of the Mac1/NOX2 pathway in mediating reactive microgliosis-generated chronic neuroinflammation and progressive neurodegeneration. Curr Opin Pharmacol.

[21] Salbach PB, Specht E, von Hodenberg E, Kossmann J, Janssen-Timmen U, Schneider WJ, Hugger P, King WC, Glomset JA, Habenicht AJ (1992). Differential low density lipoprotein receptor-dependent formation of eicosanoids in human blood-derived monocytes. Proc Natl Acad Sci USA.

[22] Qu P, Yan C, Blum JS, Kapur R, Du H (2011). Myeloid-specific expression of human lysosomal acid lipase corrects malformation and malfunction of myeloid-derived suppressor cells in lal-/- mice. J Immunol.

[23] Bozza PT, Magalhaes KG, Weller PF (2009). Leukocyte lipid bodies - Biogenesis and functions in inflammation. Biochim Biophys Acta.

[24] Radović B, Vujić N, Leopold C, Schlager S, Goeritzer M, Patankar JV, Korbelius M, Kolb D, Reindl J, Wegscheider M, Tomin T, Birner-Gruenberger R, Schittmayer M (2016). Lysosomal acid lipase regulates VLDL synthesis and insulin sensitivity in mice. Diabetologia.

[25] Buchebner M, Pfeifer T, Rathke N, Chandak PG, Lass A, Schreiber R, Kratzer A, Zimmermann R, Sattler W, Koefeler H, Fröhlich E, Kostner GM, Birner-Gruenberger R (2010). Cholesteryl ester hydrolase activity is abolished in HSL-/- macrophages but unchanged in macrophages lacking KIAA1363. J Lipid Res.

[26] Sacerdoti D, Gatta A, McGiff JC (2003). Role of cytochrome P450-dependent arachidonic acid metabolites in liver physiology and pathophysiology. Prostaglandins Other Lipid Mediat.

[27] Rosenbaum AI, Cosner CC, Mariani CJ, Maxfield FR, Wiest O, Helquist P (2010). Thiadiazole carbamates: potent inhibitors of lysosomal acid lipase and potential Niemann-Pick type C disease therapeutics. J Med Chem.

[28] Rosenbaum AI, Rujoi M, Huang AY, Du H, Grabowski GA, Maxfield FR (2009). Chemical screen to reduce sterol accumulation in Niemann-Pick C disease cells identifies novel lysosomal acid lipase inhibitors. Biochim Biophys Acta.

[29] Hamilton J, Jones I, Srivastava R, Galloway P (2012). A new method for the measurement of lysosomal acid lipase in dried blood spots using the inhibitor Lalistat 2. Clin Chim Acta.

[30] Vujic N, Schlager S, Eichmann TO, Madreiter-Sokolowski CT, Goeritzer M, Rainer S, Schauer S, Rosenberger A, Woelfler A, Doddapattar P, Zimmermann R, Hoefler G, Lass A (2016). Monoglyceride lipase deficiency modulates endocannabinoid signaling and improves plaque stability in ApoE-knockout mice. Atherosclerosis.

[31] Goeritzer M, Vujic N, Schlager S, Chandak PG, Korbelius M, Gottschalk B, Leopold C, Obrowsky S, Rainer S, Doddapattar P, Aflaki E, Wegscheider M, Sachdev V (2015). Active autophagy but not lipophagy in macrophages with defective lipolysis. Biochim Biophys Acta.

[32] Cortner JA, Coates PM, Swoboda E, Schnatz JD (1976). Genetic variation of lysosomal acid lipase. Pediatr Res.

[33] Kortz L, Dorow J, Becker S, Thiery J, Ceglarek U (2013). Fast liquid chromatography-quadrupole linear ion trap-mass spectrometry analysis of polyunsaturated fatty acids and eicosanoids in human plasma. J Chromatogr B Analyt Technol Biomed Life Sci.

[34] Sattler W, Puhl H, Hayn M, Kostner GM, Esterbauer H (1991). Determination of fatty acids in the main lipoprotein classes by capillary gas chromatography: BF3/methanol transesterification of lyophilized samples instead of Folch extraction gives higher yields. Anal Biochem.

